# Ambulatory arterial stiffness indices and target organ damage in hypertension

**DOI:** 10.1186/1471-2261-12-1

**Published:** 2012-01-27

**Authors:** Manuel Ángel Gómez-Marcos, José Ignacio Recio-Rodríguez, Ma Carmen Patino-Alonso, Leticia Gómez-Sánchez, Cristina Agudo-Conde, Marta Gómez-Sánchez, Emiliano Rodríguez-Sánchez, Luís García-Ortiz

**Affiliations:** 1 Primary Care Research Unit, La Alamedilla Health Centre, Salamanca, Spain. REDIAPP; 2 Statistics Department, University of Salamanca, Salamanca, Spain

**Keywords:** Ambulatory arterial stiffness index, home arterial stiffness index, ambulatory blood pressure monitoring, home blood pressure, target organ damage

## Abstract

**Background:**

The present study was designed to evaluate which arterial stiffness parameter - AASI or the home arterial stiffness index (HASI) - correlates best with vascular, cardiac and renal damage in hypertensive individuals.

**Methods:**

A cross-sectional study was carried out involving 258 hypertensive patients. AASI and HASI were defined as the 1-regression slope of diastolic over systolic blood pressure readings obtained from 24-hour recordings and home blood pressure over 6 days. Renal damage was evaluated by glomerular filtration rate (GFR) and microalbuminuria; vascular damage by carotid intima-media thickness (IMT), pulse wave velocity (PWV) and ankle/brachial index (ABI); and left ventricular hypertrophy by the Cornell voltage-duration product (VDP) and the Novacode index.

**Results:**

AASI and HASI were not correlated with microalbuminuria, however AASI and HASI- blood pressure variability ratio (BPVR) showed negative correlation with GRF. The Cornell PDV was positively correlated with AASI- BPVR-Sleep (r = 0.15, p < 0.05) and the left ventricular mass index with HASI-BPVR (r = 0.19, p < 0.01). Carotid IMT and PWV were positively correlated with all the parameters except the HASI, while ABI was negatively correlated with AASI and Awake-AASI. After adjusting for age, gender and 24 hours heart rate, statistical significance remains of the IMT with AASI, Awake AASI and AASI-BPVR. PWV with the AASI, Awake-AASI and Sleep-AASI. ABI with AASI and Awake-AASI. Odd Ratio to presence target organ damage was for AASI: 10.47(IC95% 1.29 to 65.34), Awake-AASI: 8.85(IC95% 1.10 to 71.04), Sleep-AASI: 2.19(IC95% 1.10 to 4.38) and AASI-BPVR-night: 4.09 (IC95% 1.12 to 14.92).

**Conclusions:**

After adjusting for age, gender and 24-hour heart, the variables that best associated with the variability of IMT, PWV and ABI were AASI and Awake-AASI, and with GFR was HASI-BPVR.

## 1.- Background

The ambulatory arterial stiffness index (AASI) is related to cardiovascular morbidity-mortality [1,2] and to the presence of associated target organ damage (TOD) in hypertensive patients [3-8]. The AASI is very useful for assessing arterial stiffness, shows a strong correlation to other classical measures such as pulse wave velocity (PWV) [9,10], and in contrast to the latter requires no special or costly equipment. However, AASI determination requires ambulatory blood pressure monitoring (ABPM), which limits its generalized use in clinical practice.

Taking into account the above, home blood pressure monitoring (HBP) also offers a range of measures of blood pressure in the usual environment of the individual, with a good correlation to cardiovascular morbidity-mortality [11] and the presence of TOD [3,6,7]; moreover, its use is less costly and more accessible, and the technique is better accepted by the patients.

The relationship between the home arterial stiffness index (HASI) and AASI has not been well established. A study has concluded that HASI may serve as a useful surrogate measure of arterial stiffness [12], while other authors consider HASI to be less closely associated to markers of arterial stiffness [13,14]. We were interested in determining whether the arterial stiffness index calculated by HASI may be an alternative to AASI in predicting TOD. If the measurements of the two parameters are similar, then HASI would have a considerable potential for application in clinical practice. If not found to be similar, we can determine whether AASI and HASI are correlated differently with other vascular, cardiac or renal lesions, and whether they can have different implications in clinical practice.

The present study was designed to evaluate which arterial stiffness parameter - AASI or the home arterial stiffness index (HASI) - correlates best with vascular, cardiac and renal damage in hypertensive individuals.

## 2.- Methods

### 2.1.- Study design and population

A cross-sectional study was carried out in a primary care setting. We consecutively included all hypertensive subjects visiting primary care clinics between January 2008 and January 2011, and referred to the research unit for the assessment of cardiovascular risk. Hypertension was diagnosed when the mean of three recordings in the clinic under basal conditions and separated in time revealed systolic blood pressure (SBP) ≥ 140 and/or diastolic blood pressure (DBP) ≥ 90 mmHg. On each visit at least two recordings were made, spaced more than one minute apart. The included patients were aged 30-80 years, and individuals with a history of cardiovascular disease (ischemic heart disease or stroke) or diabetes mellitus were excluded. The sample size to detect a minimum correlation coefficient of 0.2 with two-sided type I error rate of 5% and 80% power was estimated to be 194 individuals The sample of 258 subjects was therefore considered to be sufficient for this study, which adhered to the principles of the Declaration of Helsinki, and was approved by an independent ethics committee of Salamanca University Hospital (Spain). Written informed consent to participation in the study was obtained in all cases.

### 2.2.- Measurement

#### 2.2.1.- Office or clinical blood pressure

Office blood pressure measurement involves three measurements of SBP and DBP, using the average of the last two measurements, with a validated OMRON model M7 sphygmomanometer (Omron Health Care, Kyoto, Japan), and following the recommendations of the European Society of Hypertension [15]. The office blood pressure values used in the study were the mean values of the last two measurements.

#### 2.2.2.- Home blood pressure (HBP)

Three measurements were made in the morning (between 6:00 and 9:00 a.m.), and three in the afternoon/evening (between 18:00 and 21:00 p.m.), over a period of 7 days, with a minimum interval of one minute between measurements, and excluding the first measurement and the values corresponding to the first day of measurement [16]. Twenty-four blood pressure measurements were used to estimate HASI. The same sphygmomanometer model used to measure blood pressure in the office was employed. Each patient included in the study received an instructions sheet and a form specifically designed for recording the blood pressure values.

#### 2.2.3.- Ambulatory blood pressure monitoring (ABPM)

ABPM was performed on a day of standard activity, with an adequate cuff for the size of the patient's arm. A control system (Spacelabs 90207, Healthcare, Issaquah, Washington, USA), validated according to the protocol of the British Hypertension Society, was used [17]. The records of readings considered to be valid were ≥ 80% of the total. The monitor was programmed for obtaining blood pressure measurements every 20 min during the waking period and every 30 min during the resting period. Individual correction was made of the waking and sleeping hours reported by the patient.

#### 2.2.4.- Assessment of carotid intima-media thickness (IMT)

Carotid ultrasonography to assess IMT was performed by two investigators trained for this purpose before starting the study. A Sonosite Micromax ultrasound device paired with a 5-10 MHz multifrequency high-resolution linear transducer with Sonocal software was used for performing automatic measurements of IMT, in order to optimize reproducibility. Measurements were made of the common carotid artery after the examination of a longitudinal section of 10 mm at a distance of 1 cm from the bifurcation, performing measurements in the proximal wall, and in the distal wall in the lateral, anterior and posterior projections, following an axis perpendicular to the artery to discriminate two lines (one for the intima-blood interface and the other for the media-adventitia interface). A total of 6 measurements were obtained of the right carotid and another 6 of the left carotid. The measurements were obtained, following the recommendations of the Manheim Carotid Intima-Media Thickness Consensus [18]. The average IMT was considered abnormal if it measured 0.90 mm, or if there were atherosclerotic plaques with a diameter of 1.5 mm or a focal increase of 0.5 mm or 50% of the adjacent IMT [19].

#### 2.2.5.- Evaluation of peripheral artery involvement

This was evaluated using the ankle/brachial index (ABI). The blood pressure in the upper and lower extremities was measured using a portable Doppler system Minidop Es-100Vx (Hadeco, Inc. Arima, Miyamae-ku, Kawasaki, Japan), applying the probe at the posterior tibial artery at an angle of approximately 60° to the direction of blood flow

The ABI was calculated separately for each foot by dividing the higher of the two systolic pressures in the ankle by the higher of the two systolic pressures in the arm. TOD was considered if the ABI was lower than 0.9 [19].

#### 2.2.6.- Pulse wave velocity (PWV)

**Pulse wave velocity (PWV) **was estimated with the SphygmoCor System (AtCor Medical Pty Ltd Head Office, West Ryde, Australia), with the patient in the supine position. The pulse waves of the carotid and femoral arteries were analyzed, estimating the delay with respect to the ECG wave and calculating PWV. PWV is calculed as the ratio of the distance travelled (calculated as distance in mm of distal minus proximal, where measures are taken from the suprasternal notch to the sampling site) and the foot-to-foot time delay between the pulse waves and expressed in meters per second (m/sec). TOD was considered if the PWV was higher than 12 m/sec [19].

#### 2.2.7.- Renal assessment

Kidney damage was assessed by measuring creatinine plasma concentration; the glomerular filtration rate (GFR) was estimated by the CKD-EPI (Chronic Kidney Disease Epidemiology Collaboration) for Caucasians [20]; and proteinuria was determined from the albumin/creatinine ratio following the ESH 2007 criteria. TOD was defined as plasma creatinine 1.3 mg/100 ml or higher in men and 1.2 mg/100 ml or higher in women, and GFR below 60 ml/min or an albumin/creatinine ratio ≥ 22 mg/g in men and ≥ 31 mg/g in women [19].

#### 2.2.8.- Cardiac assessment

The electrocardiographic examination was performed using a General Electric MAC 3.500 ECG System (General Electric, Niskayuna, NY, USA) that automatically measures the voltage and duration of waves and estimates the criteria of the Cornell voltage duration product (Cornell-VDP) [21]. The left ventricular mass index (LVMI) was estimated by the Novacode equation [19,22]. TOD was defined According to the 2007 European Society of Hypertension/European Society of Cardiology Guidelines criteria [19].

#### 2.2.9.- AASI and HASI

Arterial stiffness was evaluate with ambulatory arterial stiffness index (AASI and AASI- blood pressure variability (BPVR)) and home arterial stiffness index (HASI and HASI (BPVR)). For AASI and HASI estimation, the regression slope of diastolic on systolic blood pressure was computed for each individual on the basis of 24-hour ABPM (AASI) and also HBP readings (HASI) over 6 days. AASI as well as HASI were defined as one minus the respective regression slope of DBP on SBP. AASI was also computed from waking or sleeping blood pressure. Blood pressure variability ratio (BPVR) was defined as SD (SBP)/SD(DBP), AASI (BPVR) as 1-[1/SD (SBP)/SD (DBP)] in 24-hour blood pressure [23-25], and HASI (BPVR) as 1-[1/SD (SBP)/SD (DBP)] over 6 days of HBP recording.

The individuals performing the different tests were blinded to the clinical data of the patient. All organ damage assessment measures were made within a period of 10 days.

### 2.3.- Statistical analysis

Continuous variables were expressed as the mean ± standard deviation (SD), while frequency distributions were used for qualitative variables. Mean of AASI and HASI were adjusted by age and gender based on analysis of covariance (ANCOVA). Pearson's correlation coefficient was used to estimate the relationship between quantitative variables, while the chi-squared test was used to associate qualitative variables. We performed multiple linear regression analysis using carotid IMT, PWV, ABI, Cornell PDV and glomerular filtration as dependent variables. The enter method was used in a first step to include as adjustment variables patient age, gender (male = 1; female = 0) and 24-hour heart rate, and then we included one by one the independent variables (AASI, Awake-AASI, Sleep-AASI, AASI-BPVR, AASI-BPVR-Awake, AASI-BPVR-Sleep HASI and HASI-BPVR) to avoid collinearity. Logistic regression analysis by the enter method was performed to evaluate the association between ambulatory arterial stiffness measures as independent variables, included one by one, and TOD as the dependent variable (1 with TOD and 0 without TOD), adjusted by age and gender. The data were analyzed using the SPSS version 18.0 statistical package (SPSS Inc., Chicago, Illinois, USA).

## 3.- Results

The demographic and clinical characteristics, office and ambulatory blood pressure parameters used to evaluate TOD, and the arterial stiffness indices are reported in Tables 1 and 2. The mean age was 53 years, and 60% of the patients were males. The mean 24-hour and Awake-AASI was 0.37 ± 0.06, Sleep-AASI was 0.38 ± 0.15, and HASI was 0.59 ± 0.18. Fourteen percent of the hypertensive patients showed renal damage, 21% vascular damage, and 9% left ventricular hypertrophy (LVH).

**Table 1:** General demographic and clinics characteristics in hypertensive patients.

Variables	N = 258
Age	53.3 ± 2.0
Male, n (%)	153(59.3)
Smokers, n (%)	64(24.8)
Body mass index, kg/m2	27.92 ± 3.83
Waist circumference, (cm)	95.60 ± 11.12
Total Cholesterol, (mg/dl)	208.96 ± 36.94
Tryglicerides, (mg/dl)	126.34 ± 73.35
LDL cholesterol, (mg/dl)	130.64 ± 33.00
HDL cholesterol,(mg/dl)	53.28 ± 3.01
Serum creatinine, (mg/dl)	0.90 ± 0.19
Albumin/creatinine (mgg)	14.65 ± 48.59
GFR estimated with CKD-EPI	88.62 ± 15.41
Cornell VDP (mmms)	1543.89 ± 695.93
LVMI (gm^2^) Novocode	74.85 ± 18.33
Carotid IMT (mm)	0.71 ± 0.12
PWV (m/sec)	8.65 ± 2.06
ABI	1.08 ± 0.10
AASI	0.37 ± 0.06
Awake-AASI	0.37 ± 0.06
Sleep-AASI	0.38 ± 0.15
AASI-BPVR	0.16 ± 0.16
AASI-BPVR-Awake	0.19 ± 0.20
AASI-BPVR-Sleep	0.08 ± 0.25
HASI	0.59 ± 0.18
HASI-BPVR	0.29 ± 0.24
**TOD**	94(36.4)
Renal, n(%)	37(14.3)
Vascular, n(%)	54(20.9)
Heart, n(%)	24(9.3)

Data for qualitative variables are expressed as n (%) and quantitative variables as mean ± standard deviation. LDL: Low density lipoprotein; HDL: High density lipoprotein; GFR: Glomerular filtration; CKD-EPI: Chronic Kidney Disease Epidemiology Collaboration; VDP: Voltage-duration product; LVMI: Left ventricular mass index; IMT: Intima-media thickness; ABI: Ankle/brachial index; PWV: Pulse wave velocity; AASI: Ambulatory arterial stiffness index; Awake AASI = Ambulatory arterial stiffness index in Awake time; Sleep AASI: Ambulatory arterial stiffness index in sleep time; BPVR: Blood pressure variability ratio; HASI: Home arterial stiffness index; TOD: target organ damage.

**Table 2:** Characteristics of blood pressure assessed by different methods and used antihypertensive drugs.

Variables	
Years of evolution of hypertension	7.0 ± 5.5
Subjects receiving drug treatment n(%)	101(39.1)
Patients with white coat hypertension n(%)	32(20.4)
**Drugs used in treatment**	
Diuretics n(%)	44(17.1)
ACE inhibitors n(%)	37(14.3)
Angiotensin receptor blockers n (%)	33(12.8)
Calcium channel blockers n (%)	16(6.2)
**Office BP (mmHg)**	
SBP, mm Hg	139.9 ± 16.8
DBP, mm Hg	88.3 ± 10.7
PP, mm Hg	52.2 ± 12.8
HR bpm	72.1 ± 12.9
**ABPM 24 hours (mmHg)**	
SBP, mm Hg	126.7 ± 12.8
DBP, mm Hg	78.7 ± 9.9
PP, mm Hg	47.9 ± 9.5
HR bpm	71.9 ± 10.6
SD SBP	13.8 ± 3.3
SD DBP	11.3 ± 2.4
N° blood pressure measurement	60
**Awake time ABMP (mmHg)**	
SBP, mm Hg	130.6 ± 13.3
DBP, mm Hg	82.3 ± 10.6
PP, mm Hg	48.4 ± 9.5
HR bpm	75.1 ± 11.6
SD SBP	11.9 ± 3.3
SD DBP	9.5 ± 2.5
N° blood pressure measurement	45
**Sleep time ABPM (mmHg)**	
SBP, mm Hg	115.1 ± 14.5
DBP, mm Hg	68.3 ± 9.9
PP, mm Hg	46.9 ± 10.2
HR bpm	62.6 ± 9.2
SD SBP	10.3 ± 3.5
SD DBP	9.1 ± 2.8
N° blood pressure measurement	15
**Sleep/Awake ratio SBP**	0.9 ± 0.1
**Sleep/Awake ratio DBP**	0.8 ± 0.1
**% Dipping Systolic**	11.7 ± 8.0
**HOME BP (mmHg)**	
SBP, mm Hg	127.2 ± 14.4
DBP, mm Hg	81.6 ± 9.8
PP, mm Hg	45.6 ± 10.1
HR bpm	68.4 ± 9.1
SD SBP	9.1 ± 3.4
SD DBP	6.2 ± 2.3
N° blood pressure measurement	28

Data for qualitative variables are expressed as n (%) and quantitative variables as mean ± standard deviation.

ACE: angiotensin-converting enzyme; SBP: systolic blood pressure; DBP: dyastolic blood pressure; PP: pulse pressure; HR: heart rate; bpm: beats per minute; ABPM: ambulatory blood pressure monitoring; BP: blood pressure.

Table 3 shows the correlation between ambulatory arterial stiffness measures and target organ damage indices. Age was positively correlated with all the ambulatory arterial stiffness measures, being the lowest with HASI (r = 0.13, p < 0.05) and the highest with AASI-BPVR-Awake (r = 0.45, p < 0.01). AASI shows a greater correlation with PP than HASI.. AASI and HASI not correlated with microalbuminuria and AASI and HASI-BPVR proved negative to CKD-EPI. The Cornell PDV was positively correlated with AASI-BPVR-Sleep (r = 0.15, p < 0.05), and LVMI with HASI-BPVR (r = 0.19, p < 0.01). Carotid IMT and PWV were positively correlated with all the parameters except HASI, while ABI was negatively correlated with AASI and Awake-AASI.

**Table 3:** Bivariate correlations of AASI and HASI with age and target organ damage in hypertensive patients

	AASI 24 h	Awake-AASI	Sleep-AASI	AASI-BPVR	HASI	HASI-BPVR
Age	0.42**	0.41**	0.18**	0.44**	0.13*	0.32**
Office PP, mm Hg	0.58**	0.57**	0.25**	0.42**	0.13*	0.15*
ABPM 24 h PP, mm Hg	0.85**	0.85**	0.38**	0.46**	0.21**	0.12
HOME BP PP, mm Hg	0.61**	0.60**	0.28**	0.39**	0.18**	0.16**
CKD-EPI	-0.31**	-0.30**	-0.12	-0.26**	-0.06	-0.27**
Albumin/creatinine (mgg)	0.11	0.11	-0.01	0.08	-0.02	0.05
Cornell VDP (mmms)	0.03	0.01	0.02	0.09	-0.01	0.01
LVMI (gm^2^) Novocode	0.03	0.02	0.05	0.07	0.05	0.19**
Carotid IMT (mm)	0.41**	0.39**	0.19**	0.38**	0.07	0.22**
PWV (m/seg)	0.29**	0.28**	0.25**	0.29**	0.05	0.24**
ABI	-0.13*	-0.13*	-0.04	-0.01	0.03	-0.01

AASI: Ambulatory arterial stiffness index; HASI: Home arterial stiffness index; H: Hours; Awake AASI: Ambulatory arterial -stiffness index in Awake time; Sleep-AASI: Ambulatory arterial stiffness index in sleep time; BPVR: Blood pressure variability ratio; PP: pulse pressure; ABPM: Ambulatory blood pressure monitoring; BP: Blood pressure; CKD-EPI: Chronic Kidney Disease Epidemiology Collaboration; VDP: Voltage-duration product; LVMI: Left ventricular mass index; IMT: Intima-media thickness; PWV: Pulse wave velocity; ABI: Ankle/brachial index; AASI: Ambulatory arterial stiffness index.

*p < 0.05, statistically differences, **p < 0.01, statistically differences.

The correlation of PWV with office PP was: (r = 0.32, p < 0.01); with PP ABPM 24 h (r = 0.33, p < 0.01); and with PP HOME BP (r = 0.39, p < 0.01).

The observed correlations between AASI with TOD indices were greater (p < 0.05) than those of HASI in GFR, carotid IMT, PWV and ABI. The results were not modified on excluding from the analysis those patients with white coat hypertension, or on separately analyzing those receiving drug treatment and those receiving no drug treatment.

After adjusting for age, gender and 24 hours heart rate (table 4), statistical significance remains of the IMT with AASI (β = 0.32), Awake-AASI (β = 0.29) and AASI-BPVR (β = 0.08. PWV with the AASI (β = 4.95), Awake-AASI (β = 4.57) and Sleep-AASI (β = 2.47). ABI with AASI (β = -0.28) and Awake-AASI (β = -0.28). Finally CKDEPI only with HASI-BPVR (β = -7.35) and with heart indices no ambulatory arterial stiffness measures reached statistical significance.

**Table 4:** Multiple Linear Regression Analysis: Relationship Between TOD and parameters that evaluate arterial stiffness in hypertensive patients

Variable	Not standardized β	Confidence interval 95%	*P Value*
**Dependent variable: IMT**			

AASI	0.32	0.12 to 0.52	0.00
Awake-AASI	0.29	0.08 to 0.49	0.01
Sleep-AASI	0.06	-0.01 to 0.13	0.11
AASI-BPVR	0.08	0 01 to 0.15	0.03
HASI	-0.01	-0.06 to 0.06	0.90
HASI-BPVR	0.02	-0.03 to 0.07	0.39

**Dependent variable: PWV**			

AASI	4.95	0.83 to 9.07	0.02
Awake-AASI	4.57	0.42 to 8.72	0.03
Sleep-AASI	2.47	1.10 to 3.84	0.00
AASI-BPVR	1.42	-0.09 to 2.92	0.07
HASI	-0.29	-1.54 to 0.96	0.64
HASI-BPVR	0.83	-0.14 to 1.79	0.09

**Dependent variable: ABI**			

AASI	-0.28	-0.51 to -0.04	0.02
Awake-AASI	-0.28	-0.52 to -0.04	0.02
Sleep-AASI	-0.03	-0.11 to 0.05	0.45
AASI-BPVR	-0.01	-0.09 to 0.08	0.86
HASI	0.02	-0.05 to 0.09	0.61
HASI-BPVR	0.01	-0.05 to 0.06	0.82

**Dependent variable: CKDEPI**			

AASI	-12.74	-44.31 to 18.84	0.43
Awake-AASI	-12.55	-44.33 to 19.23	0.44
Sleep-AASI	-0.85	-11.42 to 9.72	0.87
AASI-BPVR	2.73	-8.63 to 14.10	0.64
HASI	0.35	-8.94 to 9.63	0.94
HASI-BPVR	-7.35	-14.59 to -0.10	0.05

Adjusted Variables: Age; Gender: (male = 1; female = 0); 24 hours Heart rate.

Dependent variables: Carotid IMT: Carotid Intima-media thickness, PWV: pulse wave velocity, ABI: ankle/brachial index and Glomerular filtration.

Independent variable: AASI, Awake-AASI, Sleep-AASI, AASI-BPVR, HASI and HASI-BPVR.

TOD: target organ damage; CKD-EPI: Chronic Kidney Disease Epidemiology Collaboration; LVMI: Left ventricular mass index; IMT: Intima-media thickness; PWV: Pulse wave velocity; ABI: Ankle/brachial index; AASI: Ambulatory arterial stiffness index; BPVR: Blood pressure variability ratio; Awake AASI: Ambulatory arterial stiffness index in Awake time; Sleep AASI: Ambulatory arterial stiffness index in sleep time; HASI: Home arterial stiffness index.

**Figure 1:**
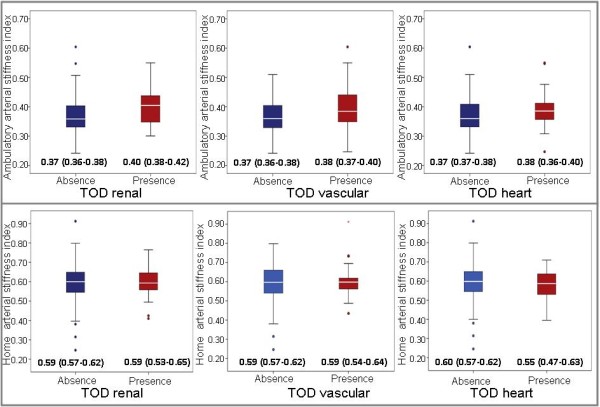
**Boxplots showing the median, 25th and 75th percentiles and maximum and minimum values of AASI and of HASI, according to the presence or absence of renal, vascular or cardiac target organ damage (TOD)**. Adjustment for age and gender. Inside the box mean (CI95%) AASI and HASI adjusted by age and gender.

**Figure 2:**
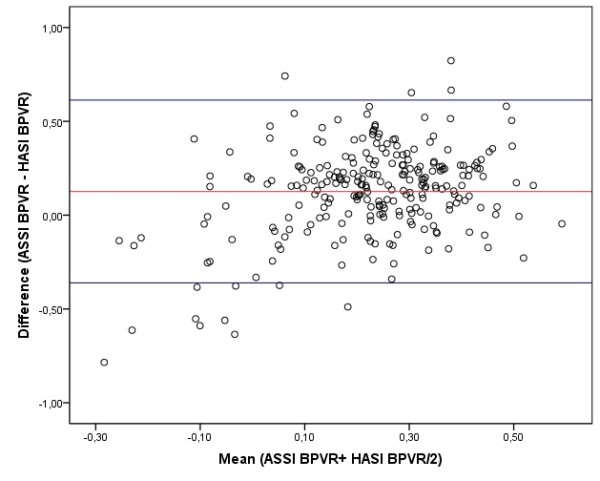
**Bland-Altman plots of Ambulatory arterial stiffness index (AASI) and Home arterial stiffness index (HASI)**.

Figure 1 shows the boxplots for AASI and HASI according to the presence or absence of renal, vascular or cardiac damage. Mean of AASI and HASI were adjusted by for age and gender. The Odds ratio (OR) of ambulatory arterial stiffness measures, adjusted by age, gender and 24 hours heart rate, to predict TOD, were for AASI: 1.83 (IC95% 1.07 to 3.22), Awake-AASI: 1.78 (IC95% 1.08 to 3.08), Sleep-AASI: 1.17 (IC95% 0.47 to 3.07) and AASI-BPVR-Sleep: 1.15 (IC95% 1.01 to 1.31). Else measures did not reached statistical significance. According to the Bland-Altman analysis the limits of intra-observer agreement was 0.13 (95%CI:-0.36 to 0.61) and the repeatability coefficients was 0.49 (Figure 2).

## 4.- Discussion

The present study shows that the behavior of the two approaches for measuring stiffness through home blood pressure monitoring and its relation to TOD differs. Thus, while HASI is not correlated to any of the parameters used to evaluate the presence of TOD in hypertensive patients, HASI-BPVR is positively correlated with IMT and PWV, and shows a negative correlation with GFR, in the same way as the AASI indices except Sleep-AASI, which shows a negative correlation with GFR. In turn, AASI-BPVR-Sleep and which shows a positively correlation with Cornell-PDV and HASI-BPVR which shows a positively correlation with LVMI.

These results indicate that stiffness arterial assessed with HASI-BPVR to detect the presence of vascular, renal or cardiac damage associated to hypertensive patients is better than HASI.

In previous studies, Qureshi et al. [12] reported mean HASI scores of 0.62 ± 0.20 in 49 hypertensive patients. Stergiou GS et al.[13], in a series of 483 treated and non-treated hypertensive subjects, reported a mean HASI score of 0.66 ± 0.17 the latter being higher than the corresponding 24-hour AASI score (0.33 ± 0.15), Awake-AASI (0.50 ± 0.18) and Sleep-AASI (0.37 ± 0.19). These results are similar to the HASI findings in our own series (0.59 ± 0.18), and differ from our 24-hour AASI score (0.37 ± 0.06) and Awake-AASI (0.37 ± 0.06) and Sleep-AASI scores (0.38 ± 0.15), which yielded similar values.

The correlations found between HASI and the AASI measures have an r-value of close to 0.18, while the values reported by Stergiou [13] were r = 0.14 for 24-hour and Awake-AASI, and r = 0.09 for Sleep-AASI. In the same way as published by other authors [12,13], positive associations are observed between AASI and HASI and patient age and pulse pressure in its different measures.

The positive association between AASI and TOD has already been reported by Leoncini et at. [4] in 188 subjects without drug treatment the risk of developing any form of TOD being found to be greater as the AASI score increases. Likewise, Garcia et al. [26], in a recent study of 554 hypertensive individuals with and without treatment, reported correlations between AASI and TOD similar to those found in our series. Our results showed a higher AASI score in the case of subjects with renal and vascular damage. The association between left ventricular hypertrophy and AASI is not clear. Schillaci et al. [27] reported a relationship between AASI and the left ventricular mass index, assessing the latter by means of echocardiography in non-treated hypertensive individuals. However, in coincidence with the observations of Leoncini et al [4], the association lost strength upon adjusting for age and gender. In our study, AASI was not related to the electrocardiographic parameters used to assess LVMI. However, we found a positive correlation between HASI (BPVR) and LVMI estimated with the Novacode index, for which we are unable to offer a clear explanation.

Our findings on evaluating renal TOD confirm those published by Ratto et al. [5] and Mulè et al. [28], who documented a negative correlation between AASI and renal damage as assessed by GFR (r = -0.25, p < 0.01; r = -0.30, p < 0.01, respectively); and by Hermans et al. [29], who estimated renal damage based on the MDRD formula. On evaluating renal damage with urinary albumin excretion, we observed no significant results, in contrast to Leoncini et al. [4], who obtained a positive association after logarithmic transformation of the albumin-creatinine index.

Schillaci et al.[27] and Jerrard-Dunne et al.[30] reported a relationship between AASI and PWV, though this correlation disappeared upon adjusting for patient age. In the present study, the association to PWV persisted after fitting for age, gender and heart rate only in the case of Sleep-AASI. The positive correlation of AASI to IMT and the negative correlation to ABI likewise confirm the results of previous studies [4]. Triantafyllidi et al. [8], in a study of 168 non-treated hypertensive subjects, obtained a correlation coefficient between AASI and IMT of r = 0.34, similar to the value recorded in our series. The association was maintained in the multiple regression model only in the case of those patients exhibiting a dipping pattern.

### Study limitations

The main limitation of our study is its cross-sectional design, which precludes the definition of a causal relationship between AASI and different parameters in evaluating TOD. Another limitation is the fact that ours was not a randomized sample, and so we cannot extrapolate the results to all hypertensive patients. In order to correctly interpret the 24-hour, Awake-AASI and Sleep-AASI values, it must be taken into account that the waking/sleeping measurements ratio is 3 - as a result of which the waking values are over-dimensioned with respect to the sleeping values.

## Conclusions

The ambulatory arterial stiffness measures, except HASI were positively correlated with IMT and PWV, and negatively correlated with glomerular filtration. After adjusting for age, gender and 24-hour heart, the variables that best associated to the variability of IMT, PWV and ABI were AASI and Awake-AASI, and with CKDEPI was HASI-BPVR. HASI-BPVR shows greater correlation with the studied target organ damage parameters than HASI.

### Perspectives

Lastly, new prospective studies would be needed in order to confirm the usefulness of HASI-BPVR in assessing arterial stiffness with home blood pressure monitoring.

## Competing interests

The authors declare that they have no competing interests.

## Authors' contributions

MAGM devised the study, designed the protocol, participated in fund raising, interpretation of results, prepared the manuscript draft and corrected the final version of the manuscript. JIRR and CAC participated in the study design, data collection and manuscript review. MCPA performed all analytical methods, interpretation of results, and manuscript review. ERS, LGS and MGS participated in the study design, interpretation of results, and manuscript review. LGO participated in the protocol design, fund raising, analysis of results, and final review of the manuscript. Finally, all authors reviewed and approved the final version of the manuscript.

## Pre-publication history

The pre-publication history for this paper can be accessed here:

http://www.biomedcentral.com/1471-2261/12/1/prepub
